# A model for a partnership of lipid transfer proteins and scramblases in membrane expansion and organelle biogenesis

**DOI:** 10.1073/pnas.2101562118

**Published:** 2021-04-13

**Authors:** Alireza Ghanbarpour, Diana P. Valverde, Thomas J. Melia, Karin M. Reinisch

**Affiliations:** ^a^Department of Cell Biology, Yale University School of Medicine, New Haven, CT 06520

**Keywords:** TMEM41B, VMP1, ATG9A, scramblase

## Abstract

The autophagy protein ATG2, proposed to transfer bulk lipid from the endoplasmic reticulum (ER) during autophagosome biogenesis, interacts with ER residents TMEM41B and VMP1 and with ATG9, in Golgi-derived vesicles that initiate autophagosome formation. In vitro assays reveal TMEM41B, VMP1, and ATG9 as scramblases. We propose a model wherein membrane expansion results from the partnership of a lipid transfer protein, moving lipids between the cytosolic leaflets of apposed organelles, and scramblases that reequilibrate the leaflets of donor and acceptor organelle membranes as lipids are depleted or augmented. TMEM41B and VMP1 are implicated broadly in lipid homeostasis and membrane dynamics processes in which their scrambling activities likely are key.

A long-standing fundamental question in cell biology is how organelles, such as the autophagosome, can form de novo. The recent discovery that ATG2, required for early steps in autophagosome formation, is a member of a class of lipid transport protein proposed to function in bulk lipid transfer suggests a model of membrane growth (1–3). Namely, ATG2 could mediate lipid transfer from the endoplasmic reticulum (ER), where most cellular lipid synthesis takes place, to the expanding isolation membrane. ATG2 localizes to contact sites where the ER and the nascent autophagosome are in close apposition (3). Based on structural studies of ATG2 and related proteins, ATG2 forms a bridge between the ER and the autophagosome with a hydrophobic channel along which lipids could flow (4). However, transfer of lipids will occur only between the cytosolic leaflets of the apposed bilayers. Left unchecked, such a process would lead to bilayer asymmetry both in the ER, where lipids are depleted, and in the autophagosome, where lipids are augmented, and ultimately to membrane destabilization. Thus, the model in which organelle expansion is supported by protein-mediated lipid transfer predicts the existence of mechanisms, such as scramblases, to reequilibrate lipids between leaflets both in the lipid donor and lipid acceptor membranes. To test this model, we biochemically characterized integral membrane proteins with reported roles in autophagosome biogenesis, finding that consistent with the model they are scramblases and, further, they interact physically with ATG2. Although unanticipated, their direct interaction with ATG2 builds confidence that these scramblases could partner with ATG2 in bulk lipid transfer.

## Results

We identified three scramblase candidates, each implicated in early autophagosome biogenesis events. ATG9A is present on Golgi-derived vesicles required to initiate autophagosome formation (5) while TMEM41B and VMP1 reside in a complex on the ER and are necessary during autophagosome expansion (6–8). Intriguingly, TMEM41B and VMP1 share a six-helix transmembrane domain also present in the DedA family of bacterial proteins, predicted half transporters with poorly understood roles in membrane homeostasis (8).

We used a well-established fluorescence-based in vitro scrambling assay (9) to assess whether TMEM41B, VMP1, or a 1:1 mixture of the two proteins scrambles phospholipids (*SI Appendix*, Fig. S1 *A* and *B*). For this assay, the proteins were overexpressed in mammalian (Expi293F) cells and purified in *n*-dodecyl-β-d-maltoside detergent, then reconstituted into liposomes containing a small percentage of nitrobenzoxadiazole (NBD)-labeled lipid. Dithionite reduces solvent-exposed NBD and quenches its fluorescence. Thus, in the absence of scrambling, a 50% reduction in fluorescence is expected as diothionite cannot access NBD in the liposome lumen. In the presence of a scramblase, NBD lipids are continuously exchanged between the leaflets of the bilayer, making all NBD accessible, so that the fluorescence reduction should be complete (>>50%) in the ideal reconstitution scenario when 100% of the liposomes incorporate the scrambling activity. Using this assay, we found that TMEM41B, VMP1, or a 1:1 mixture can scramble NBD-phosphatidylethanolamine (PE) (*SI Appendix*, Fig. S1*C*). In parallel, we used the same protocols to purify and reconstitute the well-characterized intramembranous protease GlpG into liposomes, finding that GlpG does not scramble NBD-PE under our conditions. We confirmed that the fluorescence reduction observed in the presence of TMEM41B/VMP1, TMEM41B, or VMP1, is not due to leaky liposomes that have bilayer defects, for example due to incomplete detergent removal, so that dithionite could penetrate into the liposome lumen. To this end, we prepared proteoliposomes as before, but lacking NBD lipids, and in the presence of NBD glucose. We found that the NBD glucose remains within the liposome lumen even when the liposomes are extensively dialyzed against NBD glucose-free buffer and that NBD glucose in the lumen is not affected by the addition of dithionite. We also show, using both the dithionite scrambling assay and a similar “back extraction assay” (*SI Appendix*, Fig. S1 and *SI Methods*), that lipid scrambling by TMEM41B/VMP1, TMEM41B, and VMP1 are not specific to a particular glycerolipid (*SI Appendix*, Fig. 1*D*) as both NBD-PE and NBD-phosphatidylcholine (PC) are substrates.

We used the dithionite scrambling assay to show that ATG9A also scrambles lipids, including PC, PE, and phosphatidylserine (*SI Appendix*, Fig. S1 *E–G*). Lipid scrambling by ATG9A was also recently reported by others, who additionally showed by structure-based mutational analysis that this scrambling activity is essential for autophagosome growth (10, 11).

We next explored whether ATG9A and TMEM41B/VMP1 might interact directly with the lipid transport protein ATG2A. We coexpressed 3XFLAG-tagged ATG9A and untagged ATG2A in Expi293F cells, finding that ATG2A robustly copurifies with ATG9A in an affinity purification, associating nearly stoichiometrically (*SI Appendix*, Fig. S2*A*). To assess whether ATG2A might interact with TMEM41B or VMP1, we overexpressed ATG2A in Expi293F cells, and passed the lysate over 3XFLAG-TMEM41B or -VMP1 immobilized on anti-FLAG resin. ATG2A is robustly retained by both TMEM41B and VMP1, indicating that it interacts with either protein.

ATG2A is an elongated structure proposed to interact with apposed ER and autophagosome membranes via its ends, with its N terminus at one and C-terminal portions at its other end (3). If so, ATG2A should interact with TMEM41B/VMP1 via one end and ATG9A via the other. To date, we are only able to make a soluble N-terminal, but not C-terminal fragment of ATG2A. We find that this fragment, mini-ATG2A (residues 1 to 345), associates with TMEM41B and VMP1 but not ATG9A in interaction experiments similar to the ones described above for the full-length protein (*SI Appendix*, Fig. S2 *A* and *B*). To increase confidence in our results, we also used mini-ATG2A in flotation assays with either protein-free liposomes or liposomes reconstituted with ATG9A, TMEM41B, VMP1, or TMEM41B/VMP1 (*SI Appendix*, Fig. S2*C*). In this experiment, mini-ATG2A was incubated with liposomes, and then liposomes and any associated mini-ATG2A were separated from unbound protein by density gradient centrifugation. We found that mini-ATG2A associates robustly only with liposomes reconstituted with TMEM41B, VMP1, or TMEM41B/VMP1 but not empty or ATG9A-containing liposomes. (Intact ATG2A associates nonspecifically with liposomes and so flotation assays with the full-length protein were not informative.) These data are consistent with the “bridge” model (*SI Appendix*, Fig. S2*D*). Although a direct interaction between the lipid transport protein and scramblases should not be necessary for bulk lipid transport in principle, such interactions between ATG2A and ATG9A or TMEM41B/VMP1 suggest there may be inherent advantages to coupling scramblase and transport activities spatially.

## Discussion

We consider plausible that the autophagosome could grow even from a single ATG9-containing vesicle, acting as a seed membrane. In this scenario, ATG2 allows lipid transport from the ER to the seeding vesicle, with TMEM41B and VMP1 reequilibrating the leaflets of the ER as lipids are extracted and ATG9 in the “seed” scrambling ER-derived lipids as they are delivered (see also refs. 10, 11). This would allow for the expansion of the membrane surface area of the seed even while the volume of contents enclosed within the membrane remains relatively constant. Expansion in this way would result in a double membrane structure like the autophagosome. High membrane curvature is energetically costly, so the nascent autophagosome would not form a double membrane sheet, with an expansive high curvature circumference, but would instead spontaneously curl up into a cup-shaped structure (12), with a much smaller high curvature area, as observed in the maturing autophagosome. Interestingly, a protein structurally related to ATG2, VPS13, plays a role in prospore formation in yeast and acrosome formation in humans, where both the prospore and acrosome are also cup-shaped double-membrane structures initiated from a small number of vesicles (13, 14). Autophagosomes, prospores, and acrosomes may arise via similar mechanisms involving a partnership of lipid transfer proteins and scramblases. Of note, human VPS13A was reported to form a complex with a predicted scramblase, XK (15).

VMP1 and TMEM41B have both been implicated in multiple processes other than autophagy, all potentially associated in some way with membrane dynamics. The discovery that VMP1 and TMEM41B are scramblases can explain much of the apparently disparate biology associated with these proteins. First, both VMP1 and TMEM41B are implicated in lipid homeostasis as depletion of either leads to the accumulation of neutral lipids into oversized cytoplasmic lipid droplets (7, 8, 16). Both proteins are enriched at organelle-organelle contact sites along with key lipid-synthesis machinery (16–18), and thus are ideally situated to facilitate redistribution of lipids from the ER via soluble lipid transfer proteins localized at contact sites. The defects in lipid homeostasis may reflect challenges in lipid-synthesis and neutral lipid consumption when efficient efflux of phospholipids is hindered. Further, VMP1 was also recently shown as essential for lipoprotein production (19). Lipoprotein assembly takes place in the ER lumen, where apolipoproteins are assembled with lipids asymmetrically derived from the luminal leaflet of the ER membrane prior to packaging into vesicles for transport out of the cell. Depletion of VMP1 leads to the production of amorphous lipoprotein-like particles that suggest partial budding into both the cytoplasmic and luminal leaflets simultaneously, and a complete loss of effective secretion. This suggests that analogous to coupling of scramblases and cytoplasmic lipid transport proteins, there is a comparable functional coupling of scramblase activity and the effective building of lipoprotein particles. And lastly, the proteins were recently reported as essential for corona- and flavirus replication, although no mechanisms were identified (20, 21). These viruses form replication compartments derived from the ER membrane. Coronavirus replication compartments in particular, described as double membrane spherical structures, resemble autophagosomes. We speculate that the replication compartments might form de novo, like the autophagosome, involving a partnership between lipid transfer proteins and scramblases.

## Supplementary Material

Supplementary File

## Data Availability

All study data are included in the article and/or *SI Appendix*.
