# Supplementary material for: A model for a partnership of lipid transfer proteins and scramblases in membrane expansion and organelle biogenesis
Source: Proc Natl Acad Sci U S A. 2021 Apr 13;118(16):e2101562118. doi: 10.1073/pnas.2101562118 (PMC8072408; doi:10.1073/pnas.2101562118)
Supplement: Supplementary File [file pnas.2101562118.sapp.pdf]

A model for a partnership of lipid transfer proteins and scramblases in membrane expansion and organelle biogenesis.

Alireza Ghanbarpour*, Diana P. Valverde*, Thomas J. Melia†, Karin M. Reinisch†

Department of Cell Biology, Yale University School of Medicine, New Haven, CT 06520.

Supplementary information – Figure S1, **Figure S2**, and **Materials and Methods**

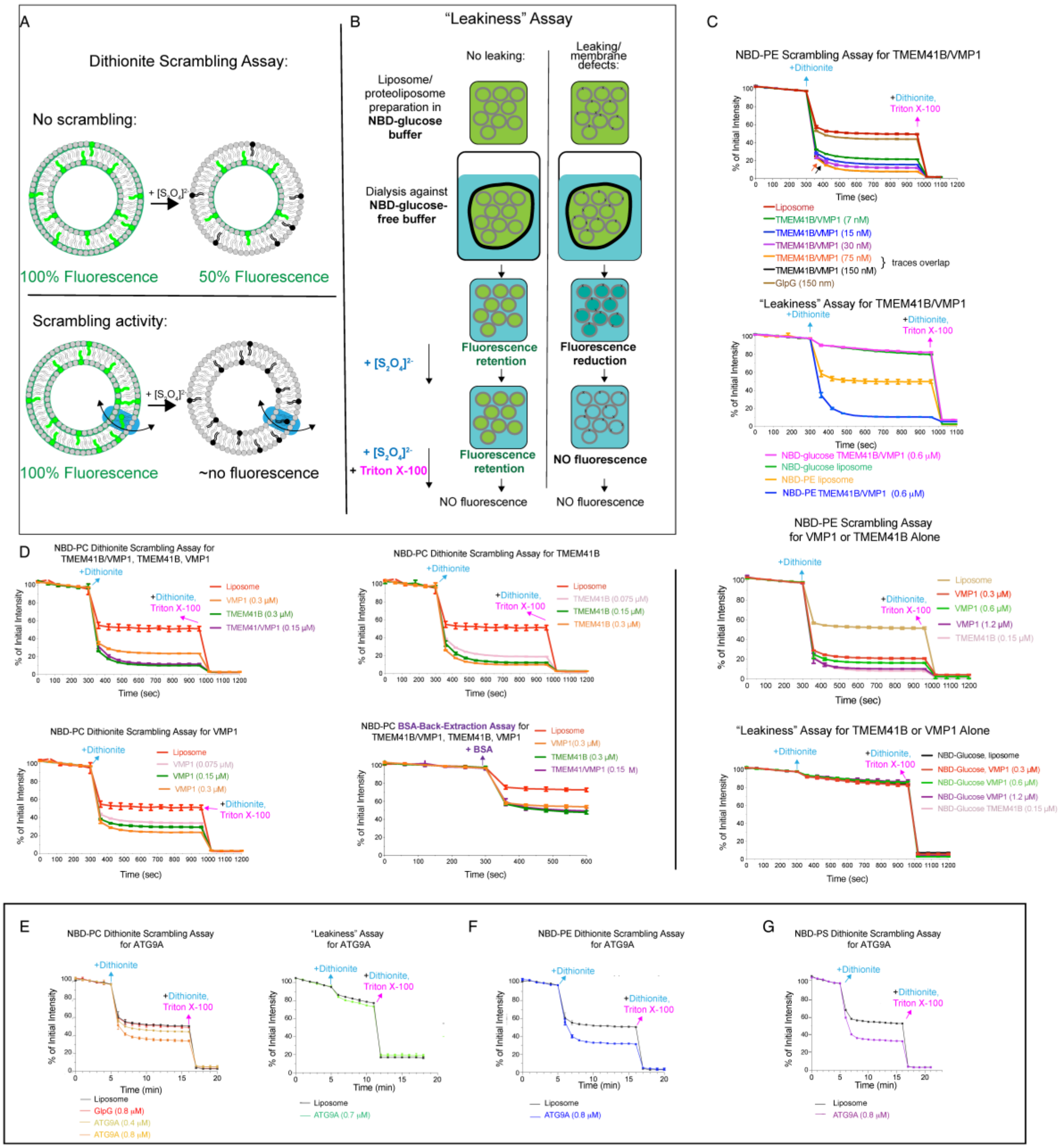

Figure S1. TMEM41B, VMP1, and ATG9A scramble lipids in vitro. (A, B) Schematics for the scrambling and "leakiness" assays. (C) 1:1 mixtures of TMEM41B and VMP1 or TMEM41B or VMP1 alone, can scramble NBD-PE. Scrambling is not observed in liposomes reconstituted with the control protein GlpG or in empty liposomes. Reconstitution into liposomes is more efficient when the proteins are added at higher concentrations, resulting in near total reduction of fluorescence. (D) TMEM41B/VMP1, TMEM41B and VMP1 also scramble NBD-PC. Additionally, a bovine serum albumin (BSA) "back extraction" assay in which BSA instead of dithionite was added to liposomes/proteoliposomes, shows that TMEM41B/VMP or TMEM41B or VMP1 alone scramble PC. In this assay, the NBD-PC located in the outer leaflet of the liposome is extracted and quenched by fatty-acid free BSA instead of being reduced by dithionite. BSA binding reduces NBD-PC fluorescence by half versus NBD-PC fluorescence in liposomes. Thus, fluorescence would be reduced to 75% of initial levels in the absence of a scramblase and to ~50% if there is scrambling. All experiments were repeated at least three times; SD indicated. (E-G) Scrambling assays for Atg9. The reduction in fluorescence for ATG9 (to ~35% of initial levels) is less than for TMEM41B or VMP1; this does not imply less scrambling activity for ATG9 but rather less efficient reconstitution of functional ATG9 into liposomes. Protein concentrations throughout refer to the monomeric forms of the proteins.

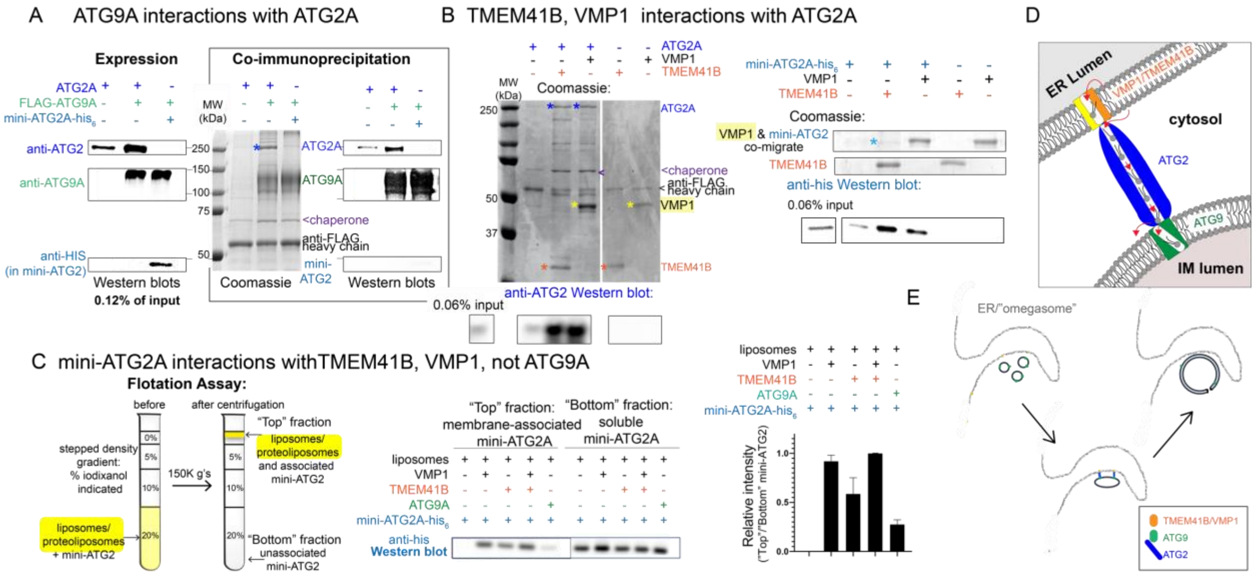

Figure S2. The lipid transporter ATG2A interacts with ATG9, TMEM41B, and VMP1 and could function as a lipid transport bridge. (A) 3XFLAG-ATG9A was co-expressed with either untagged intact ATG2A or mini-ATG2A-his$_6$ (residues 1-345). Then 3XFLAG-ATG9A was bound to anti-FLAG resin along with associated proteins. ATG9A co-purifies with full length ATG2A but not with mini-ATG2A. (B) 3XFLAG-TMEM41B or -VMP1 were bound to anti-FLAG resin incubated with cell lysates containing either untagged ATG2A or mini-ATG2A-his$_6$, and washed. Both TMEM41B and VMP1 robustly pull down ATG2A and interact, though more weakly, with mini-ATG2A. All experiments in A-B were repeated at least three times; representative Coomassie stained gels and western blots are shown. (C) In the flotation assay, mixtures of liposomes or proteoliposomes and mini-ATG2A-his$_6$ were loaded onto a density gradient and centrifuged. Liposomes/proteoliposomes and associated proteins float at the top, whereas soluble proteins remain at the bottom of the gradient. Top and bottom fractions were analyzed for mini-ATG2A by SDS/PAGE and western blotting. Mini-ATG2 associates with proteoliposomes containing TMEM41B, VMP1, or their mixture, but not empty or ATG9A-containing liposomes, consistent with interactions in A-B. The experiment was repeated three times. Quantification, with SD indicated. (D) A model for ATG2A-mediated lipid transport, in which one end of ATG2A associates with a scramblase complex comprising TMEM41B/VMP1 at the ER, and the other end associates with ATG9A in the nascent autophagosome, allowing lipids to flow from the ER to the autophagosome. TMEM41B/VMP1 equilibrates the two leaflets of the ER membrane following lipid extraction from the cytosolic leaflet, and ATG9A equilibrates the leaflets of the isolation membrane following lipid delivery to the cytosolic leaflet, allowing for isolation membrane expansion. (E) ATG2 and scramblases could partner to expand an ATG9-containing vesicle into a cup-shaped double membrane like the autophagosome.

MATERIALS

All the lipids including POPC (Cat.#850457C), POPE (Cat.#850757C), DOPC (Cat.# 850375), liver PE (Cat.#840026C), POPS (Cat.#840034P), cholesterol (Cat.#700000), NBD-PE (Cat.#810153C), NBD-PC (Cat.#810122), and NBD-PS (Cat.#810198) were purchased from Avanti Polar Lipids. NBD-glucose was purchased from Abcam (Cat. #186689-07-6) and DDM from GoldBioTech (Cat.#DDM25). Bio-Beads^{TM} SM2 Adsorbent Media was purchased from BIO-RAD (Cat.#152-3920), and anti-FLAG M2 resin was from Sigma Aldrich (Cat.#A2220). 6x-His Tag Monoclonal Antibody (HIS.H8; Cat.#MA1-21315) and secondary antibody goat anti-mouse IgG (Cat.# 152-3920) were purchased from ThermoFisher Scientific. The western blot was visualized using clarity western ECL substrate (BIO-RAD, cat. # 170-5060). EDTA-free Roche cOmplete protease inhibitor cocktail (Cat.#4693159001) and Optiprep density gradient medium (Cat.# D1556) were purchased from Sigma Aldrich.

METHODS

Plasmids

The coding sequence of human TMEM41B, VMP1, and ATG9A were PCR amplified from human cDNA library and subcloned into pCMV-10 with an N-terminal 3xFLAG tag and preScission cleavage site. Sequences for untagged- and N-terminally 3xFlag-tagged ATG2A and C-terminally hexahistidine tagged mini-ATG2A (residues 1-345 followed by a 25 amino acid linker) were subcloned into pCMV-10, as described in (1). Glpg expression plasmid was gifted by the laboratory of Y. Ha (2).

Expression and Purification.

TMEM41B and VMP1 for scramblase and pull-down assays. Constructs encoding TMEM41B or VMP1 were transfected into Expi293F cells (Thermo Fisher Scientific) according to manufacturer instructions. The cells were collected 48 hours after transfection, flash frozen in liquid N_2, and stored at -80°C. Then cells were thawed at room temperature, resuspended in Buffer A (500mM NaCl, 20mM Tris.HCl, pH=8.0, 1mM TCEP.HCl, 10% glycerol, 1X protease inhibitor cocktail), and lysed using a Dounce homogenizer (10 passes). To solubilize the proteins, powdered DDM was added to the lysate (1% final concentration w/v), and the lysate was gently agitated in the cold room for 90 minutes. Solubilized fractions were separated from cell debris by centrifugation (100,000g), and supernatant was transferred via gravity flow to anti-FLAG M2 resin (Sigma Aldrich), pre-equilibrated with Buffer B (Buffer A,0.02% DDM), and washed with buffer B. To remove chaperone, resin was incubated with 1mM $MgCl_2$, 2.5mM ATP at 4 °C overnight. Bound proteins were further washed with 10 bed volumes of buffer B, then eluted using 0.2µg/ml 3xFlag peptide in Buffer B. The proteins were concentrated in Amicon 0.5 ml concentrators (10KDa cutoffs, UFC501024) and quantified by Coomassie blue staining using BSA standards.

ATG9A for scramblase assays. ATG9A was purified in a similar manner as TMEM41B, and VMP1 but using Buffer C (50 mM HEPES pH 8.0, 500 mM NaCl, 1 mM TCEP, 10% glycerol) and Buffer D (Buffer C, 0.02% DDM).

GlpG (control) for scramblase assays was purified as described in (2), except that we used DDM instead of DM throughout.

ATG9A and ATG2A/mini-ATG2A-his$_6$ co-expression and co-immunoprecipitation assays. DNA for FLAG-tagged ATG9A and either untagged ATG2A or mini-ATG2A-his$_6$ were mixed at a ratio of 1:1.5 and transfected into Expi293F cells according to manufacturer instructions. Cells were harvested 38 hours post transfection and co-immunoprecipitation experiments were performed immediately after harvest. The

pellet was resuspended in lysis Buffer E (25 mM HEPES pH 7.4, 400 mM NaCl, 10% glycerol, 1mM DTT) supplemented with protease inhibitor cocktail. To solubilize any complexes, DDM was added to a final concentration of 0.5% (w/v), and cells were homogenized by Dounce homogenizer (10 passes). The lysate was agitated for 2 hours at 4 °C, and the solubilized portion was isolated by ultra-centrifugation (100,000 g's) at 4 °C for 1 hour. The supernatant was incubated with anti-FLAG beads pre-equilibrated in Buffer F (25 mM HEPES pH 7.4, 400 mM NaCl, 10% glycerol, 1mM DTT, 0.02% DDM, 2x protease inhibitor cocktail) for batch binding and agitated for 2 hours at 4° C. After incubation, the beads were transferred to a column and then washed with five bed volumes of Buffer F. Chaperone was removed by washing with five further bed volumes of Buffer F supplemented with 2.5 mM ATP and 5 mM $MgCl_2$. Beads were further washed with five additional bed volumes of Buffer F supplemented with protease inhibitor cocktail, and samples were taken for SDS-PAGE and western blot analysis.

Cell lysates overexpressing either ATG2 or mini-ATG2A for interaction experiments with TMEM41B, VMP1. ATG2A or mini-ATG2A-his_6 were transfected into Expi293F cells (Thermo Fisher Scientific) according to manufacturer instructions. The cells were collected 65h hours after transfection, flash frozen in liquid N_2, and stored at -80°C. Then the cells were thawed at room temperature, resuspended in Buffer E supplemented with 1X protease inhibitor cocktail, and lysed using a Dounce homogenizer (10 passes) followed by 5 cycles of freeze-thawing. Then DDM was added to a final concentration of 0.02% prior to incubation with TMEM41B and VMP1 immobilized on anti-FLAG M2 resin.

Liposome preparation

For TMEM41B, VMP1 scrambling assays. POPC (90% total lipid by weight), POPE (9.5%), and NBD-lipid (0.5%, NBD-PE or NBD-PC) were solubilized in chloroform, then dried under a N_2 stream and then vacuum. The resulting lipid film was resuspended in buffer G (200mM NaCl, 50mM HEPES, pH 7.6) to generate a 10.5 mM lipid stock. The mixture was incubated at 37°C for 10 minutes. Then the resuspended lipids were freeze-thawed for ten cycles. The lipid mixture was extruded 30 times using a 400 nm polycarbonate filter.

For ATG9A scrambling assays. 59% DOPC, 20% Liver PE, 5% POPS, 15% Cholesterol, and 1% NBD-labeled lipids (NBD-PS, PE, or PC) were dissolved in chloroform, then dried under a N_2 stream and further under vacuum. The resulting lipid film was hydrated in Buffer H (50 mM HEPES pH 8.0, 500 mM NaCl, and 1 mM TCEP) to produce a 5.25 mM lipid stock concentration. The mixture was then incubated at 37 °C for 1 hour, vortexed to homogeneity, and freeze-thawed for ten cycles, vortexing in between. Liposomes were then extruded through a 400 nm polycarbonate filter 31 times.

Proteoliposome preparation

Proteoliposomes were prepared as described in (3,4). Liposomes at final lipid concentration of 5.25 and 4.2 mM for TMEM41B/VMP1 and ATG9A, respectively, in 250 μL total volume were destabilized by addition of Triton-X-100 to a final concentration determined by the swelling assay as described in (5). The final Triton-concentration was 7 mM for TMEM41B/VMP1 and 8.9 mM for ATG9A. After 2-3 hours of destabilization at room temperature, detergent solubilized proteins (ATG9A, TMEM41B, VMP1, or TMEM41B and VMP1) were added and the samples were gently rotated for an hour. The detergent was removed in three steps using pre-washed biobeads: following a first addition of biobeads (20 mg), the sample was incubated at room temperature for an hour, then another aliquot of biobeads (20mg) was added and the sample was rotated at room temperature for two more hours. Finally, the sample was pipetted into a new tube containing fresh biobeads (40mg) and rotated at 4°C overnight. Biobeads were removed by pipetting, and the sample was dialyzed against buffer G for TMEM41B or VMP1 or Buffer H for ATG9A for two days at 4°C.

GlpG used as a control in both the TMEM41B/VMP1 and ATG9A scrambling assays was incorporated into liposomes in the same way as TMEM41B, VMP1 or ATG9A.

Scramblase Assays

The scramblase assay was performed at 30°C in 96-well plates, with 100-µl reaction volumes of liposomes/proteoliposomes (~200 µM final lipid concentration) prepared as described above. To assess scrambling, NBD fluorescence after addition of dithionite (to 5 mM) was monitored (excitation at 460 nm, emission at 538 nm) using the Synergy H1 Hybrid Multi-Mode Reader (BioTek). Finally, additional diothionite (5 mM) and Triton X-100 (0.5%) were added. The Triton X-100 dissolves the liposomes, allowing complete reduction of all the NBD.

The 1-myristoyl-2-C6-NBD-PC bovine serum albumin (BSA) back extraction assay for TMEM41B/VMP1 was carried out similarly, as described in (6), except that BSA (3mg/ml) was added to liposomes/proteoliposomes instead of dithionite. BSA extracts NBD-PC from the outer leaflet of liposomes, resulting in a 50% reduction of NBD-fluorescence in the extracted lipid (versus 100% reduction with dithionite).

A similar protocol was used for the NBD-glucose leakiness assay (6), except that no NBD-lipids were incorporated into the liposomes or proteoliposomes. Instead, NBD-glucose (12.6 µM) was added to the buffer during the destabilization step.

Flotation Assay with mini-ATG2A.

Liposomes and proteoliposomes, which incorporated ATG9A, TMEM41B, VMP1, or a TMEM41B/VMP1 mixture (0.15µM) were prepared as described above. After final biobead addition, mini-ATG2A (0.15µM) was added to the liposomes/proteoliposomes (80 µL final volume) and incubated on ice for 1 hour. This sample was mixed with an equal volume of 40% iodixanol density gradient solution to bring the final concentration of iodixanol to 20%. Then 10%, 5%, and 0% iodixanol solution in buffer G were added from bottom to top. After ultracentrifugation for 1 hour (~150,000 g), samples of free protein were extracted from the bottom and liposome-associated proteins from the top of the gradient; the fraction containing liposomes was visible due to the incorporation of NBD-lipids in the liposomes. Samples were analyzed by SDS-PAGE and western blotting.

Pull-down experiments for ATG2A and TMEM41B or VMP1. TMEM41B and VMP1 were purified as above using anti-FLAG resin. A 1:1 slurry of resin, either lacking "bait" protein or with TMEM41B or VMP bound, and buffer B (100µL total) were transferred to a 5ml gravity column. ATG2A or mini-ATG2A-his$_6$ lysate were passed over the column by gravity flow over the course of 2 hours. The resin was washed with 20 volumes of buffer I (0.02%DDM, 400 mM NaCl, 20mM HEPES, 1mM DTT, 10% (v/v) glycerol, pH=7.4). Sample was loaded onto gels, resolved by SDS/PAGE, and analyzed by Coomassie Blue staining and western blotting.

Methods References

1. Valverde DP*, et al.* (2019) ATG2 transports lipids to promote autophagosome biogenesis. *J Cell Biol* 218(6):1787-1798.
2. Wang Y, Zhang Y, & Ha Y (2006) Crystal structure of a rhomboid family intramembrane protease. *Nature* 444(7116):179-180.
3. Jensen MS, Costa S, Gunther-Pomorski T, & Lopez-Marques RL (2016) Cell-Based Lipid Flippase Assay Employing Fluorescent Lipid Derivatives. *Methods Mol Biol* 1377:371-382.
4. Marek M & Gunther-Pomorski T (2016) Assay of Flippase Activity in Proteoliposomes Using Fluorescent Lipid Derivatives. *Methods Mol Biol* 1377:181-191.
5. Ploier B & Menon AK (2016) A Fluorescence-based Assay of Phospholipid Scramblase Activity. *J Vis Exp* (115).

6. Verchere A, Broutin I, & Picard M (2017) Reconstitution of Membrane Proteins in Liposomes. *Methods Mol Biol* 1635:259-282.
